# Supplementary material for: Myoferlin Contributes to the Metastatic Phenotype of Pancreatic Cancer Cells by Enhancing Their Migratory Capacity through the Control of Oxidative Phosphorylation
Source: Cancers (Basel). 2019 Jun 19;11(6):853. doi: 10.3390/cancers11060853 (PMC6628295; doi:10.3390/cancers11060853)
Supplement: Supplementary file 1 [file cancers-11-00853-s001.pdf]

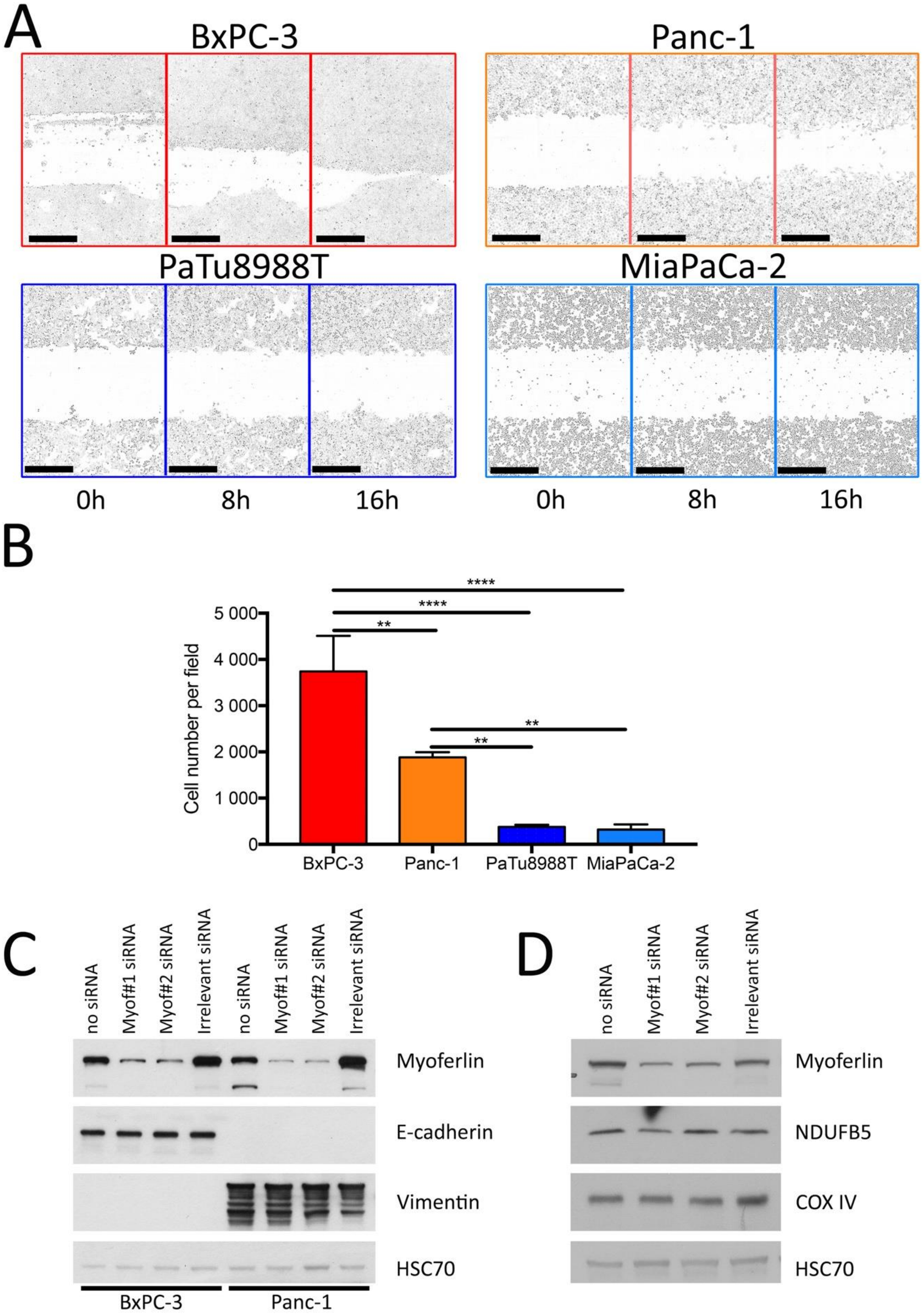

Figure S1. Myoferlin abundance correlates with pancreatic cell migratory abilities. (**A**) Representative images of two-dimension migration kinetic assay in BxPC-3, Panc-1, PaTu8988T and MiaPaCa-2 cell lines. (**B**) Quantification of migrating PDAC cell lines in the lower compartment of Boyden's chamber. (**C**) Abundance of E-cadherin and vimentin in BxPC-3 and Panc-1 cells silenced for myoferlin. (**D**) Abundance of NDUFB5 and COXIV in Panc-1 cells

silenced for myoferlin. One representative experiment out of three is illustrated. Each data point represents mean ±SD, n = 3. ****$P < 0.0001$, **$P < 0.01$.

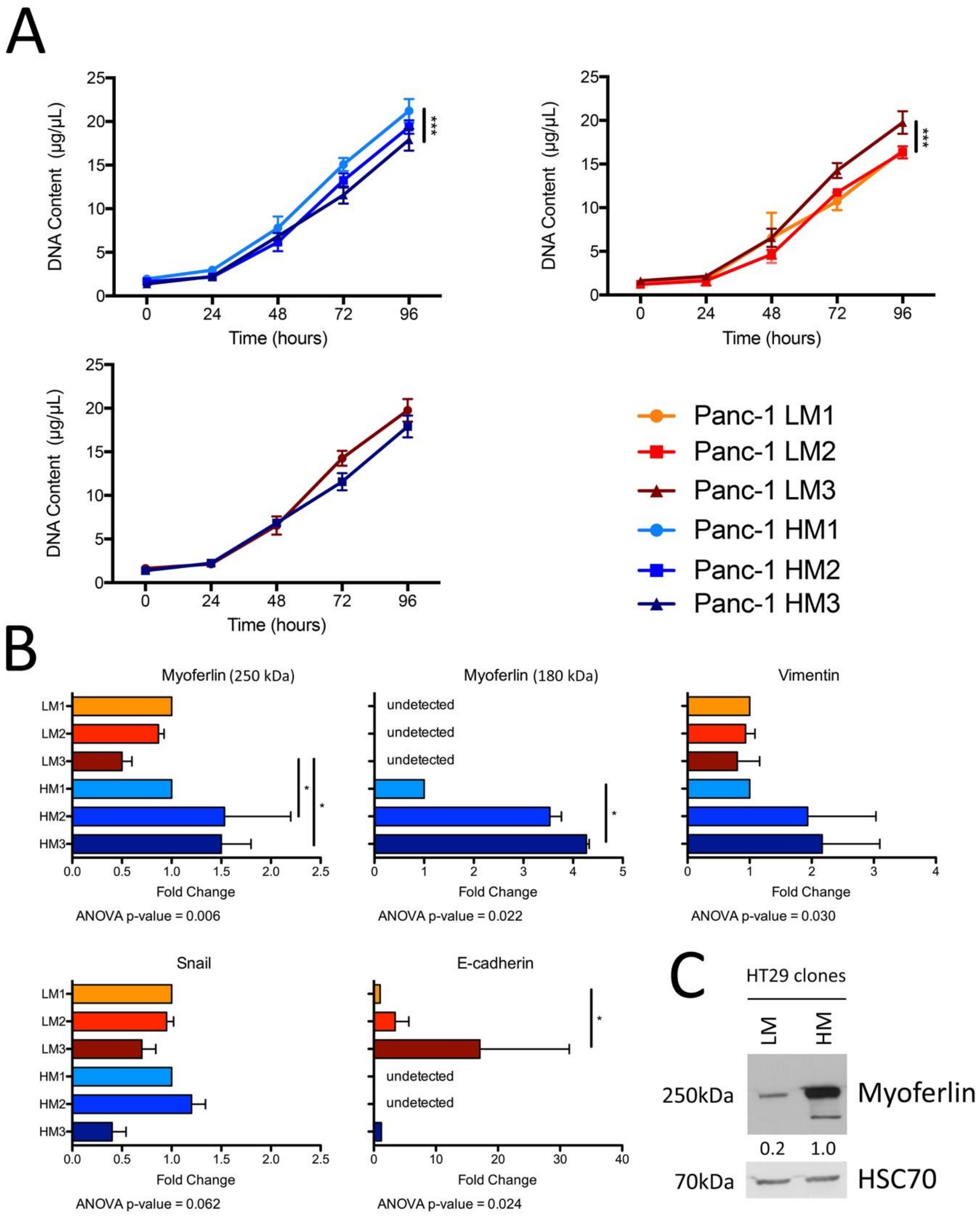

Figure S2. Myoferlin is overexpressed in cells with high metastatic potential. (**A**) Cell growth of Panc-1 clones with low (LM) or high metastatic (HM) potential assayed by Hoechst incorporation. (**B**) Myoferlin, vimentin, snail and E-cadherin western-blot relative quantification. Comparisons were performed by non-parametric Kruskal-Wallis ANOVA followed by a Dunn's multiple comparison analysis. (**C**) Myoferlin abundance in HT29 clones with low (LM) or high metastatic (HM) potential. HSC70 was used as an internal loading control. Each data point represents mean ±SD, n = 3. ***$P < 0.001$, *$P < 0.05$.

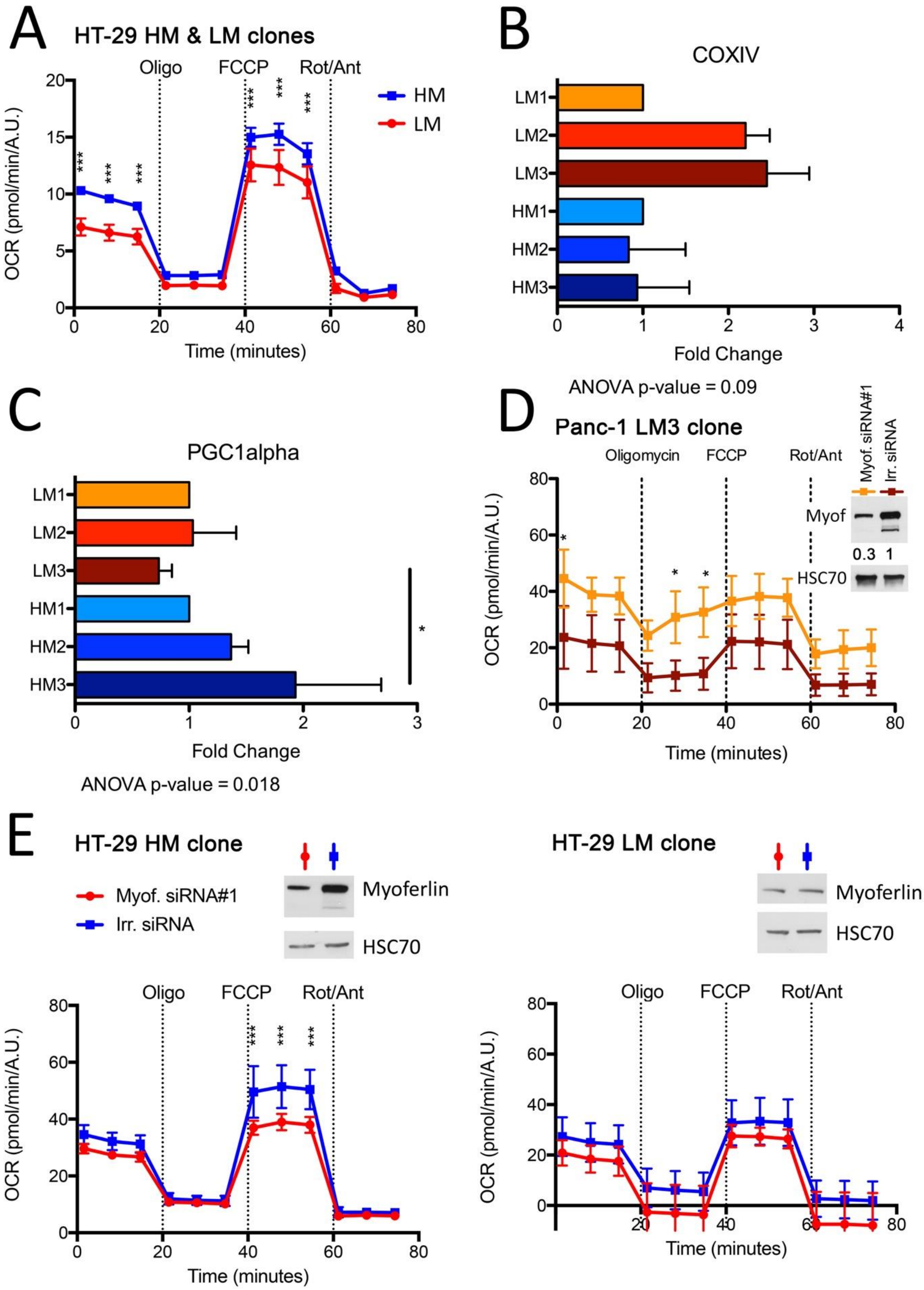

Figure S3. Myoferlin increases OXPHOS activity in HM clones. Kinetic oxygen consumption rate (OCR) of (**A**) HT29 clones (LM & HM), (**B–C**) COXIV or PGC1-a western-blot relative quantification. Comparisons were performed by non-parametric Kruskal-Wallis ANOVA followed by a Dunn's multiple comparison analysis. (**D**) Panc-1 LM3 clone silenced for myoferlin. (**E**) HT29 HM or LM clones silenced for myoferlin. OCR was recorded in

response to oligomycin (oligo, 1 μM), FCCP (1.0 μM), rotenone and antimycin A mix (Rot/Ant, 0.5 μM each). Upon assay completion, cells were methanol/acetone fixed, and cell number was evaluated using Hoechst incorporation (arbitrary unit, A.U.). Each data point represents mean ±SD, n = 3. ***$P < 0.001$, *$P < 0.05$.
